# A Rare Case of Primary Laryngeal Synovial Sarcoma, Challenges in Diagnosis and Management in a Resource-Limited Setup: A Case Report

**DOI:** 10.1155/crot/6112967

**Published:** 2025-07-24

**Authors:** Mikiyas Olani, Mesele Bogale, Suleman Essa, Amanuel Damie, Eyerusalem Fekade, Sosna Ngusie

**Affiliations:** ^1^Department of Otolaryngology-Head and Neck Surgery, College of Health Sciences, Addis Ababa University, Addis Ababa, Ethiopia; ^2^Department of Pathology, College of Health Sciences, Addis Ababa University, Addis Ababa, Ethiopia; ^3^Department of Pathology, Oncopatho Diagnostic Center, Addis Ababa, Ethiopia; ^4^Department of Internal Medicine, Yekatit 12 Medical College, Addis Ababa, Ethiopia

**Keywords:** case report, head and neck sarcoma, laryngeal synovial sarcoma, larynx sarcoma, supraglottic sarcoma, synovial sarcoma

## Abstract

**Introduction:** Sarcomas account for less than 1% of malignant tumors of the larynx. Synovial sarcomas account for 5%–7% of all soft tissue sarcomas and 0.1% of sarcomas in the head and neck region.

**Clinical Report:** A 45-year-old patient presented with hoarseness of voice and breathing difficulty. Contrast-enhanced computed tomography showed a well-defined mass originating from the laryngeal surface of the epiglottis, obstructing the supraglottis, for which emergency tracheostomy was done. Pathology confirmed monophasic synovial sarcoma. He underwent a supraglottic partial laryngectomy with complete removal of the tumor. The patient is on a 6-month postoperative course, with monthly follow-up, and there is no sign of recurrence.

**Discussion:** Clinical diagnosis can be challenging, as patients often present with ill-defined symptoms in the throat and larynx, which may delay diagnosis. The current approach relies on immunohistochemistry analysis for diagnostic purposes, and imaging is generally used to define the tumor location and extent and to rule out other tumors. Most reported studies put surgery as a first-line mode of management, and adjuvant radiotherapy is currently advocated, as it is associated with better overall survival. Synovial sarcoma is considered a high-grade tumor, although head and neck subsites tend to have a slightly better prognosis. Tumor size of more than 5 cm and higher tumor stage are associated with poor overall survival.

**Conclusion:** Synovial sarcoma of the larynx is a rare clinical entity, and every case should be examined individually.

## 1. Introduction

Mesenchymal tumors in the larynx are uncommon, and sarcomas account for less than 1% of malignant tumors of the larynx. Chondrosarcoma is the most common histologic type in the larynx [1]. Synovial sarcoma of the larynx is a rare entity, and to the best of our knowledge, so far, there are under 40 cases of laryngeal synovial sarcomas reported worldwide, with the clinic-pathologic characteristics and management still not conclusively stated [2, 3]. We report a case of a 45-year-old man with synovial sarcoma of the larynx.

## 2. Case Presentation

**Figure 1:**
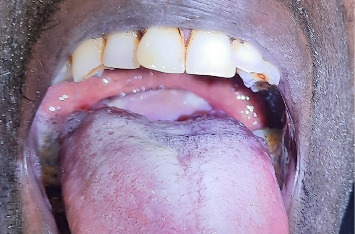
Oral examination reveals a pale mass in the oropharynx pushing tongue base and soft palate.

A 45-year-old male presented with a history of shortness of breath of 3 months' duration. Associated with this, he had a muffling of voice and dysphagia to solid foods. On oral examination, he had a pale mass visible via and narrowing of the oropharynx (Figure 1). On flexible laryngoscopy, there was a large globular mass covering the supraglottic larynx and hypopharynx. The mass seems to originate over the laryngeal surface of the epiglottis with a broad base.

**Figure 2:**
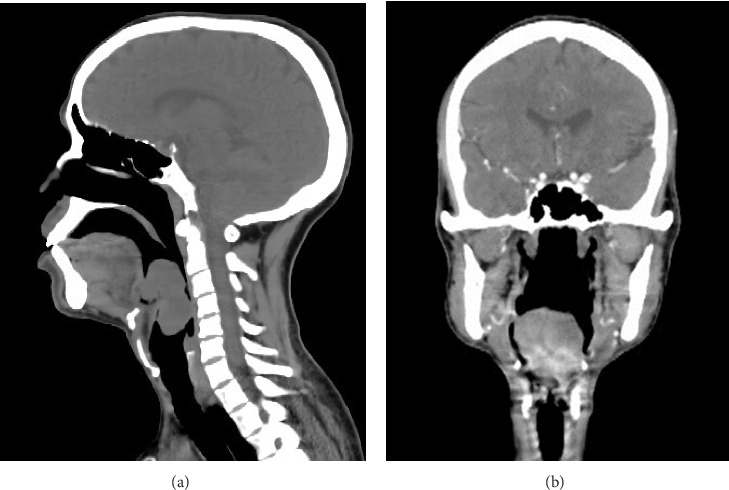
(a) Plain sagittal CT scan showing a well-defined sessile mass originating at laryngeal surface of epiglottis. (b) Coronal postcontrast image shows heterogeneous enhancement.

Contrast-enhanced CT scan was done at the time, suggestive of a pedunculated isodense mass arising from the epiglottic tip measuring 4.3 cm by 5.2 cm by 5.2 cm (Figures 2(a) and 2(b)). There is no sign of infiltration into the pre-epiglottic space or tongue base. There was no extralaryngeal extension and no lymph nodes identified.

**Figure 3:**
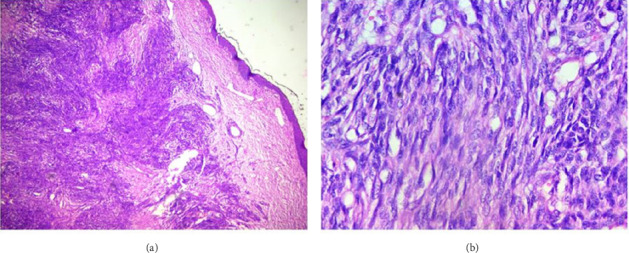
H & E: (a) Low-power view (40x) of the tumor with short fascicles of spindle cells under a bland stratified squamous epithelium. (b) High-power view (400x) of the tumor displaying the relatively monotonous spindle cell nature of the tumor with infrequent mitotic activity.

**Figure 4:**
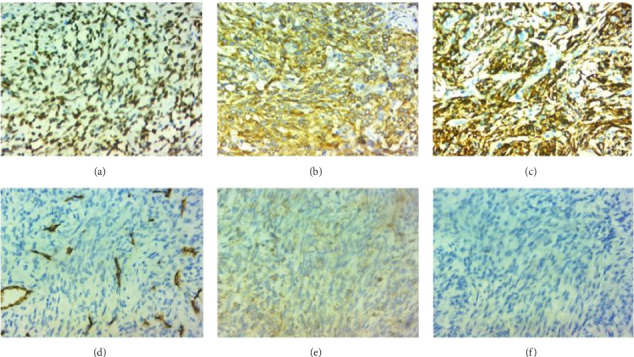
IHC: The tumor cells are positive for TLE1 (a), BCL2 (b), and CD56 (c). The tumor cells are negative for CD34 (d), STAT6 (e), and Desmin (f).

The patient's condition worsened, and he had impending upper airway obstruction for which open tracheostomy was done on an emergency basis, and direct laryngoscopy examination and biopsy were done on the mass. The histology result suggested a spindle cell tumor (Figure 3). Immunohistochemistry was suggested for better characterization. Result came with the finding of tumor being diffusely positive for TLE1, CD56, and BCL2 and negative for STAT6, CD34, and Desmin, suggestive of synovial sarcoma (Figure 4). Further molecular study for SYT-SSX protein was suggested to confirm the diagnosis but was not done because it is not available in the country.

**Figure 5:**
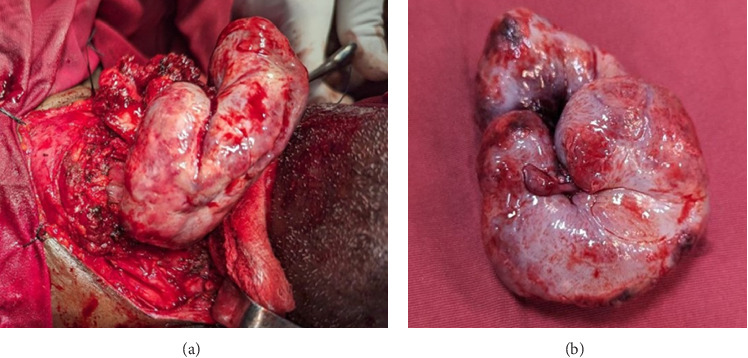
(a) Intraoperative image showing as the mass is delivered via the neck. (b) Excised fist shaped globular mass.

A multidisciplinary team consisting of a head and neck surgeon, a radiologist, and a clinical oncologist decided on upfront surgery followed by adjuvant radiotherapy. The patient underwent elective surgery through an open approach, with supraglottic partial laryngectomy done. A tumor measuring 6.3 cm by 8.5 cm was resected (Figure 5).

The postoperative course was smooth with no complications. He had intact laryngeal function and was decannulated after a 1-week hospital stay. The postoperative histopathologic report is suggestive of a monophasic synovial sarcoma, intermediate grade, with all surgical margins being free of tumor. The patient could not get postoperative radiotherapy right away, as the waiting list for radiotherapy service is long.

## 3. Discussion

The term synovial sarcoma is of historical importance since the cell of origin for the tumor is understood to be not from synovium [4, 5]. The term carcinosarcoma was proposed to show the biphasic nature of the tumor, but despite this, the original term synovial is still being used by convention [5]. Sarcomas are rare in the larynx, and especially synovial sarcoma is an even rarer diagnosis, with under 40 cases reported worldwide [6]. Unlike other soft tissue sarcomas, it preferentially affects the young group of the population with a mean age of 32 and with a male preponderence [6, 7]. Our patient is a 45-year-old male, which makes him a typical age and sex group to be affected.

The clinical presentation of a patient with laryngeal synovial sarcoma is nonspecific and mostly due to mass effect. Most common presentations are hoarseness of voice, dyspnea, dysphagia, breathing difficulty, neck swelling, and pain [3, 8]. Our patient had hoarseness of voice, dysphagia, and breathing difficulty.

Synovial sarcomas of the larynx can be misleading on imaging due to their benign-looking nature. There is no distinctive feature to differentiate them from other soft tissue masses of the larynx on CT or MRI, and diagnosis relies on the pathologic report. Imaging can be used to delineate tumor origin and extent, identify the presence of lymphadenopathy, and sometimes to rule out tumors having typical features like paragangliomas [8–10].

The histologic subtype of laryngeal synovial sarcomas is predominantly biphasic in more than 80% of reported cases. There are no reported cases of undifferentiated histology in the larynx. It is difficult to diagnose synovial sarcoma from other soft tissue sarcomas based on histology alone, especially those with monophasic and undifferentiated morphology [7, 11, 12]. Demonstration of translocation t (X; 18) by molecular tests like fluorescent in situ hybridization is considered as gold standard for diagnosis, although it has practical limitations because of unavailability in our clinical setup. Immunohistochemical markers like TLE1, BCL2, and CD56 are used to diagnose synovial sarcoma [11, 12]. Diffuse staining for TLE1 is a consistent feature of synovial sarcoma, which is used to differentiate it from other soft tissue sarcomas and is an important part of immunohistochemical panel [11]. In our case, the tumor was of monophasic histology and was diffusely positive for TLE1, Bcl2, and CD56 immunostaining.

Surgery is the mainstay of treatment in the largest series of cases reported so far, with some patients additionally treated with adjuvant radiotherapy [3, 6]. Although there is no direct comparison so far, head and neck synovial sarcoma seems to have a better prognosis than other subsites, with median overall survival at 2, 5, and 10 years being 77%, 66%, and 53%, respectively [13, 14]. Tumor size greater than 5 cm and stage at presentation were associated with poor overall survival in head and neck synovial sarcomas [13, 15]. High FDG uptake on preoperative PET/CT was reported to be associated with worse overall outcome [16]. Adjuvant radiotherapy is associated with better overall survival [13, 15]. The plan for our patient is to receive adjuvant radiotherapy, and he is currently on the waiting list at the radiotherapy clinic.

## 4. Conclusion

There is a lack of clear recommendations on the standard management and follow-up of patients with laryngeal synovial sarcomas. An individualized approach with involvement of a multidisciplinary team is essential to give the best possible care utilizing expertise. Current evidence suggests that immunohistochemistry and molecular tests are diagnostic modalities of choice. Surgery followed by adjuvant radiotherapy is associated with better overall survival in this group of patients. There is a significant risk of recurrence, and postoperative follow-up is mandatory.

## Data Availability

Data are available on request from the authors.
